# A rare case of neonatal meningoencephalitis from *Paenibacillus thiaminolyticus*

**DOI:** 10.1099/acmi.0.000430

**Published:** 2022-12-05

**Authors:** Laura S. Smallcomb, Terry C. Dixon, Kamran N. Azad, Whitney E. Marvin

**Affiliations:** ^1^​ PGY-4 Department of Medicine and Pediatrics, Medical University of South Carolina, Charleston, SC, SJCH, 10 McClennan Banks Dr. MSC 915. Charleston, SC 29425, USA; ^2^​ Department of Pediatrics. Division of Pediatric Infectious Diseases, Medical University of South Carolina, Charleston, SC, 96 Jonathan Lucas St., Rm 428C CSB. Charleston, SC 29425, USA; ^3^​ Quest Diagnostics, 33608 Ortega Highway, San Juan Capistrano, CA 92675, USA; ^4^​ Department of Pediatrics, Division of Pediatric Critical Care. Medical University of South Carolina, Charleston, SC, 125 Doughty Street MSC 917. Charleston, SC 29425, USA

**Keywords:** neonate, meningitis, encephalitis, *Paenibacillus thiaminolyticus*, meropenem

## Abstract

*
Paenibacillus* infections can be life threatening and are being reported with increasing incidence. There are only a few case reports of infections and are mainly described in patients who are immunocompromised, injection drug users, or those with prosthetic devices. Due to improved testing and identification, it appears that these infections may not be as rare as once perceived. We present a case of a 16-day-old term neonate who presented with status epilepticus and was found to have *Paenibacillus thiaminolyticus* meningoencephalitis. The patient was successfully treated with a combination of ampicillin and ceftazidime then meropenem. To our knowledge, this is the first reported case of an infant in the United States who survived this serious invasive infection. We also present an option for therapy given the difficulty treating invasive intracranial infections.

## Introduction

Cases of human infection by *Paenibacillus* species are incredibly rare and tend to occur in immunocompromised individuals, injection drug users, or those with prosthetic devices [1]. These facultative anerobic, endospore-forming, rod-shaped bacteria have been identified in bloodstream, skin and soft tissue, and central nervous system (CNS) infections [1, 2]. Much of what we understand about this genus comes from agricultural data, and the impact of human infection is limited to case reports and animal studies. In fact, case reports of CNS infection with *Paenibacillus thiaminolyticus* had been previously undescribed until the past 2 years. Here we describe a severe case of meningoencephalitis and ventriculitis with *P. thiaminolyticus* that was identified as the causative organism in cerebrospinal fluid (CSF).

## Case report

A 16-day-old Caucasian male presented to a rural coastal emergency department in the Southeastern United States with poor feeding and fever of 102 degrees Fahrenheit (38.9 degrees Celsius) that started on the day of arrival. Blood, urine, and CSF cultures were obtained. The CSF analysis was significant for 2392 white blood cells (WBC)/µl with 80 % neutrophils, protein 126 mg/dl, glucose 55 mg/dl, and 0 red blood cells (RBC)/µl. The CSF Gram-stain was negative while the Thioglycollate Broth reported Gram-negative bacilli without further comment on quantity. He was started on ampicillin (75 mg/kg every 6 hours) and gentamicin (2 mg/kg every 8 hours) and admitted to a general paediatric ward for monitoring.

Past medical history was noncontributory. He was delivered at 39 weeks via repeat caesarean section and his mother had full prenatal care throughout without reported illness. His prenatal, perinatal, and post-natal courses were unremarkable. He was fed soy-formula, but his family’s water source and sterilization techniques are unknown. There is one dog in their home and no other exposure to domestic or farm animals, and no travel since birth.

### Diagnosis and treatment

Within 24 hours of admission to the rural hospital, he began having episodes of decreased responsiveness, dilated pupils, and apnea with desaturations concerning for seizure activity. He was then transferred to a paediatric intensive care unit where he was treated with phenobarbital, midazolam, and levetiracetam; due to the sedative effects of the antiepileptics he had decreased respiratory drive and required intubation. He was transferred to our tertiary care centre at 18 days of life (DOL) for consultation with paediatric neurology and specialized monitoring. One initial blood culture grew *Staphylococcus epidermidis* but was considered a likely contaminant as his other blood cultures were negative.

Upon arrival to our facility, he was placed on continuous electroencephalogram (EEG), which showed subclinical seizures that were further treated with phenobarbital. Antibiotic regimen was continued all intravenously and expanded to ampicillin (75 mg/kg every 6 hours), ceftazidime (50 mg/kg every 8 hours), and acyclovir (20 mg/kg every 8 hours). His neurological examination was concerning as he had only right extremity movement and infrequent eye opening. An MRI was obtained that showed increased T1 signal intensity in the right frontal and parietal white matter that was suspicious for blood products, and hyperintensity of diffusion weighted imaging (DWI) in the right thalamus and corona radiata, as well as the left frontal lobe, all representing acute infarction.

Due to sustained fever, he underwent a repeat lumbar puncture on DOL 20 which had a 3448 WBC/µl with 64 % neutrophils, 7 % lymphocytes, and 26 % monocytes/macrophages, protein 471 mg/dl, glucose 1 m/dl, and 20 RBCs/µl. His procalcitonin and his C-reactive protein (CRP) were not markedly elevated on admission, and down trended throughout his course, <0.83 n/ml and <4 mg/dl respectively.

Unfortunately, the initial CSF bacterial isolate was unable to be identified at the first institution. The isolate from Thioglycollate Broth culture was sent to Quest Diagnostics. The repeat CSF culture remained negative. Acyclovir was discontinued when Herpes Simplex Virus (HSV) CSF polymerase chain reaction (PCR) was negative.

He was extubated on DOL 22 and slowly weaned off respiratory support; he had slow improvement in his neurological examination and was starting to move all extremities until he had seizure recurrence on DOL 27, which was successfully treated with phenobarbital bolus and increased dosing. Given this clinical change and continued fevers in the setting of a rise in the WBC to 18.75 K/µl, the decision was made to transition to meropenem (40 mg/ kg every 8 hours) to cover for possible resistant organisms. He had multiple repeat blood and urine cultures that remained negative.

**Fig. 1.**
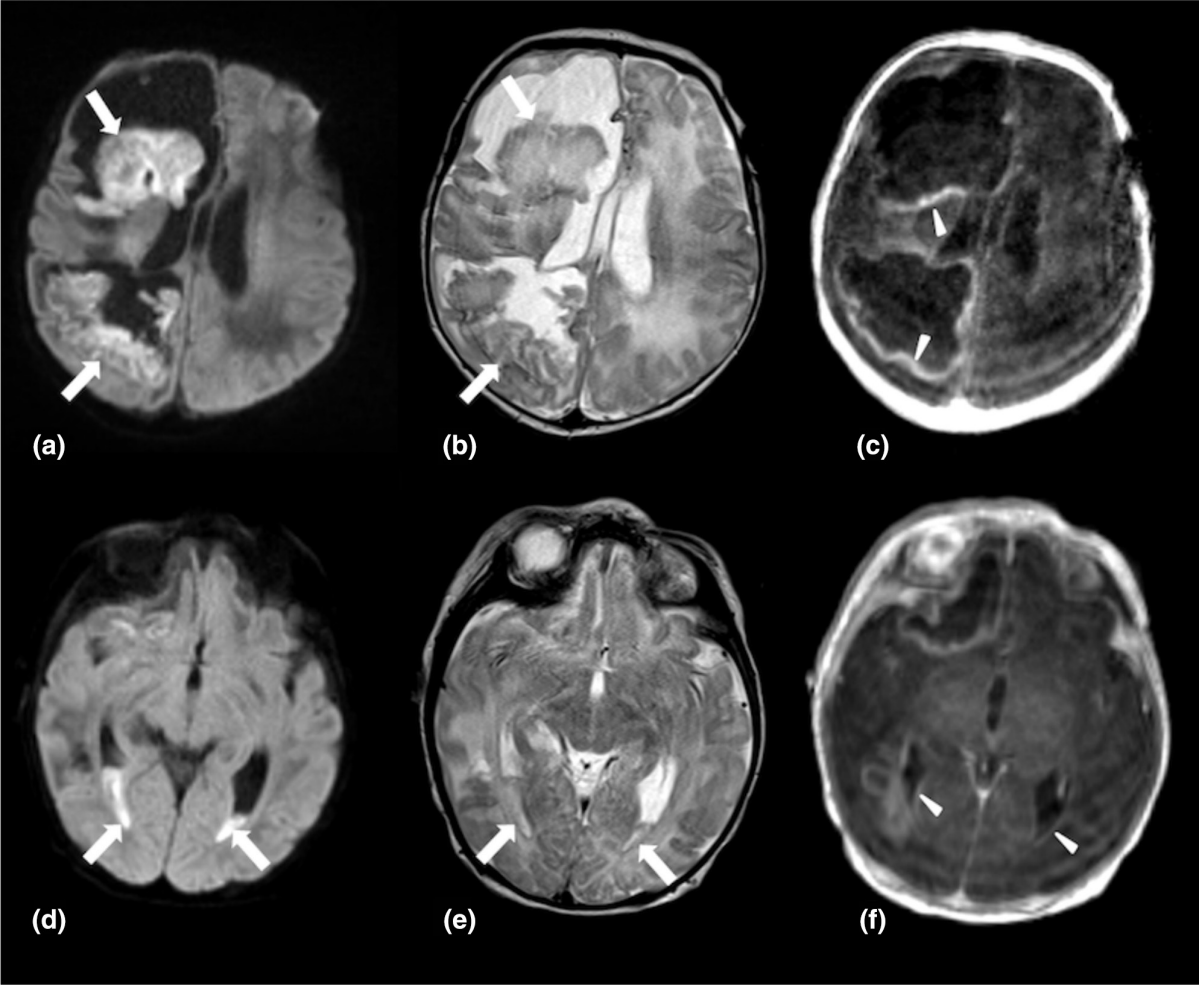
a and b, Diffusion sequence (**a**) and T2 (**b**) weighted sequences with arrows pointing to pus within in the abscesses. (c), T1 post-contrast sequence with arrowheads pointing to enhancement of the abscess walls. (d) and (e), Diffusion sequence (**d**) and T2 (**e**) sequences with arrows pointing to pus within the ventricles. (f), T1 post-contrast sequence with arrowheads pointing to enhancement of the ependymal surfaces of the ventricles.

Frequent repeat MRIs were performed. He had development of ventriculitis with purulence in the lateral ventricles, increased cystic degeneration and midline shift, progressive encephalomalacia, and new areas of restricted diffusion representing evolving areas of infarction (Fig. 1). Given the radiologic progression and recurrence of seizures, his meropenem was transitioned to continuous infusion (5 mg/kg per hour) on DOL 29, to allow for greater time over minimum inhibitory concentration (MIC) and improve efficacy on DOL 29.

His course was further complicated by development of sinus venous thrombosis (right transverse/sigmoid); in consultation with our haematology service the decision was made to forgo anticoagulation, given the high risk of life-threatening intracranial haemorrhage. Arterial imaging of the head and neck was done to help explain the predominantly unilateral nature of his infection and were normal. On DOL 29 a drain was placed into his right cystic cavity due to increasing midline shift. This drain allowed for serial CSF analysis.

The CSF isolate from Thioglycollate Broth was submitted to Quest Diagnostics, San Juan Capistrano, CA, on trypticase soy agar with 5 % sheep blood. Isolate was sub-cultured onto appropriate media and incubated accordingly to obtain fresh culture for identification and antimicrobial susceptibility testing. Identification was performed using a Bruker Daltonics MALDI-TOF (matrix-assisted laser desorption/ionization-time of flight) Biotyper. Sample preparation for MALDI-TOF protein analysis was carried out by using the 70 % formic acid extended direct protocol recommended by Bruker Daltonics. Protein spectra were analysed, and scores of 2.15 and 2.13 were obtained for *P. thiaminolyticus* (acceptable ≥2.000) using an in-house developed database. The identified pathogen (from CSF obtained on DOL 16 prior to antibiotic therapy) was shared with the primary care team on DOL 31. He was started on thiamine supplementation on DOL 32.

**Fig. 2.**
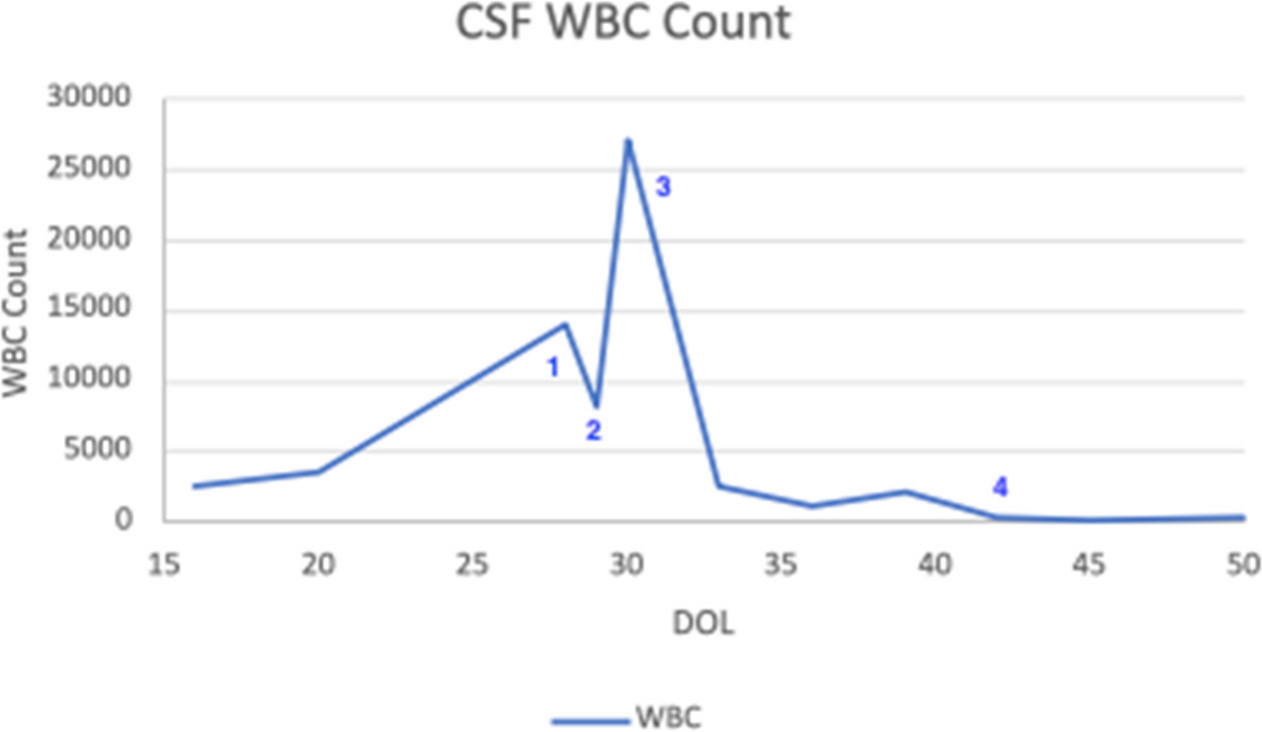
Trend of white blood cell count in the CSF (K µl^−1^) with important events. 1) Meropenem started; 2) Transition to continuous meropenem; 3) Identification of *P. thiaminolyticus *; 4) Sensitivities obtained.

Susceptibility testing was performed using Thermo Fisher Scientific, Remel, Trek Sensititre Gram-positive (GPALL3F) panel (Cleveland, OH), and minimum inhibitory concentration (MIC) in mcg/ml was interpreted and reported for chloramphenicol (≤2, susceptible), trimethoprim-sulfamethoxazole (≤2, susceptible), and vancomycin (≤4, susceptible) according to Clinical and Laboratory Standards Institute (CLSI) M45-A3 guidelines for Bacillus species (not *B *. anthracis) and related genera. Susceptibility result was received by the primary care team on DOL 42 (Table 1). He had demonstrated improvement in neurological examination and WBC in his CSF since transition to continuous meropenem, so the decision was made to continue this therapy and not add or broaden coverage (Fig. 2).

**Table 1.** Susceptibility testing from Quest Diagnostics with mean inhibitory concentration (MIC) via antibiotic gradient diffusion testing

Antibiotic	MIC (in µg/ml)	
Chloramphenicol	<2	Susceptible
Trimethoprim/Sulfamethoxazole	<2	Susceptible
Vancomycin	4	Susceptible

### Outcome and follow-up

His cystic drain was transitioned to an external ventricular drain and he eventually required an endoscopic third ventriculostomy on DOL 58 for continued hydrocephalus. Immunology specialists evaluated him and didn’t find immune dysfunction including a phagocytic defect, chronic granulomatous disease, leucocyte adhesion deficiency, or other complement-mediated dysfunction.

He completed 6 weeks of intravenous meropenem and had spontaneous and near normal movement of all extremities by discharge. He was able to take oral feeds with steady weight gain and was sent home with referral to outpatient therapies. Unfortunately, at 5 months of life, he was diagnosed with infantile spasms, but these were able to be controlled with traditional therapy. After consistent therapies as an outpatient, he is now developmentally appropriate for age at 18 months of life.

## Discussion

*Paenibacillus* species were formerly classified as Bacillus but were reclassified based on genomic data in 1991 due to a unique 16S rRNA sequence [3]. *Paenibacillus* has also been described as Gram-positive, variable, or negative which may have resulted in the initial CSF result in our patient of being Gram-negative [4]. *P. thiaminolyticus* has been linked to cerebrocortical necrosis (polioencephalomalacia) in sheep and cattle, likely due to production of the extracellular enzyme thiaminase I, leading to thiamine cleavage and overall depletion. In fact, administration of a thiamine antagonist, amprolium, has caused the characteristic brain lesions of polioencephalomalacia with microscopic astrocyte oedema in sheep [5].

Very few case reports of *P. thiaminolyticus* infection exist and were often presumed to be due to skin contamination of sterile sites [1]. Some include an infection from an indwelling dialysis catheter with associated bacteremia in an elderly patient, a neonate from a post-mortem blood culture, and an infected abdominal wall hematoma [1, 6, 7]. The neonatal case presented in cardiac arrest that later identified *P. thiaminolyticus* from a post-mortem blood culture [1]. They also had autopsy findings of intracranial infection though CSF studies were obtained late after antibiotic administration and failed to grow *P. thiaminolyticus *. Prior to this, other *Paenibacillus* species have been occasionally isolated from CSF but *P. thiaminolyticus* was not perceived to be a worrisome pathogen.

A study conducted in Uganda sampled CSF at the time of surgery from 100 consecutive cases of hydrocephalus in infants, including 64 with postinfectious hydrocephalus and 36 with non-post-infectious hydrocephalus [2]. They performed genomic testing for bacterial, fungal, and parasitic DNA, DNA and RNA sequencing for viral identification, and obtained bacterial cultures. From those with postinfectious hydrocephalus, 23 of those were found to have *P. thiaminolyticus* based on genomic assembly. The patients with *P. thiaminolyticus* presented in higher frequency with seizures and were more likely to have CT findings of intraventricular debris, calcifications, and loculations and brain abscesses than those with infectious hydrocephalus from other sources [2].

The true virulence of this organism remains unclear, though it is possible that it is more common than previously described. Further analysis of the Paulson data may suggest a geographic phenomenon or a particularly virulent strain as noted by the near complete (15/16) lethality in all mice infected [8]. *P. thiaminolyticus* also proved to be difficult to grow in culture as they were only able to isolate it three times from the 600 CSF cultures; the remainder were identified through genomic reassembly. This may have also been confounded due to the use of fresh frozen CSF and prior antibiotic administration.

Based on limited *in vitro* antimicrobial susceptibility data, it seems this organism is sensitive to common antibiotics, including gentamicin and vancomycin [9] . However, given the predilection towards loculation and calcifications, penetration of antibiotics to achieve adequate bactericidal concentrations can be a challenge. This is why we chose to treat our patient with continuous meropenem and given our positive clinical response the antibiotics were not changed back to reflect the reported sensitivities [10].

This case suggests *P. thiaminolyticus* has the potential to be severely pathogenic and fatal. It is however important to note that when *P. thiaminolyticus* is recovered in cultures, contamination and true infection are possibilities and clinical correlation is required. We feel that our patient’s extensive necrosis is in line with the previously reported cases of *P. thiaminolyticus* infection and thus was not a contaminant [1, 3]. We suspect the inability to recover the organism in subsequent cultures was secondary to pretreatment with antibiotics, though the antibiotics he received were not specifically tested for during susceptibility testing.

The fact that the majority of our patient’s left hemisphere was largely spared is what resulted in such a favourable recovery after an invasive infection. It remains unclear why nearly half his brain was preserved in this case. Given the prevalence found in the Ugandan study, perhaps *P. thiaminolyticus* is an underrecognized pathogen in cases of severe meningitis presenting with abscesses or cystic destruction, particularly given the lack of culture growth on traditional media. Use of MALDI-TOF MS may allow for appropriate and faster identification. Fortunately, it is often reported to respond to antibiotics that are commonly used to empirically treat meningitis. However, novel isolation methods in the laboratory would be beneficial to assure appropriate treatment and for diagnostic and prognostication purposes. More research will be required to assess if thiamine supplementation improves outcomes though benefits of administration likely outweigh the risk.

## Conclusions

This case, along with several other recently published case reports, demonstrate the potential pathogenicity of *Paenibacillus thiaminolyticus *, including the ability to cause intracranial infection.

Given its possible predilection for forming abscesses, continuous meropenem may enhance penetration and time above the MIC to improve bactericidal capability.

Due to the difficulty growing in culture, and Gram-variable nature, a high index of suspicion is required, improved testing capabilities with the ability to identify this infection more rapidly, may improve diagnosis and treatment.
